# Discovery of a readily heterologously expressed Rubisco from the deep sea with potential for CO_2_ capture

**DOI:** 10.1186/s40643-021-00439-6

**Published:** 2021-09-07

**Authors:** Junli Zhang, Guoxia Liu, Alonso I. Carvajal, Robert H. Wilson, Zhen Cai, Yin Li

**Affiliations:** 1 CAS Key Laboratory of Microbial Physiological and Metabolic Engineering, State Key Laboratory of Microbial Resources, Institute of Microbiology, Chinese Academy of Sciences, Beijing, 100101 China; 2 University of the Chinese Academy of Sciences, Beijing, China; 3 Department of Cellular Biochemistry, Max Planck Institute of Biochemistry, 82152 Martinsried, Germany; 4 Department of Chemistry, Massachusetts Institute of Technology, Cambridge, MA 02139 USA

**Keywords:** Rubisco, *Riftia pachyptila* endosymbiont, Form II, Hexamer, CO_2_ capture in vivo

## Abstract

Ribulose-1,5-bisphosphate carboxylase/oxygenase (Rubisco), the key CO_2_-fixing enzyme in photosynthesis, is notorious for its low carboxylation. We report a highly active and assembly-competent Form II Rubisco from the endosymbiont of a deep-sea tubeworm *Riftia pachyptila* (RPE Rubisco), which shows a 50.5% higher carboxylation efficiency than that of a high functioning Rubisco from *Synechococcus* sp. PCC7002 (7002 Rubisco). It is a simpler hexamer with three pairs of large subunit homodimers around a central threefold symmetry axis. Compared with 7002 Rubisco, it showed a 3.6-fold higher carbon capture efficiency in vivo using a designed CO_2_ capture model. The simple structure, high carboxylation efficiency, easy heterologous soluble expression/assembly make RPE Rubisco a ready-to-deploy enzyme for CO_2_ capture that does not require complex co-expression of chaperones. The chemosynthetic CO_2_ fixation machinery of chemolithoautotrophs, CO_2_-fixing endosymbionts, may be more efficient than previously realized with great potential for next-generation microbial CO_2_ sequestration platforms.

**Supplementary Information:**

The online version contains supplementary material available at 10.1186/s40643-021-00439-6.

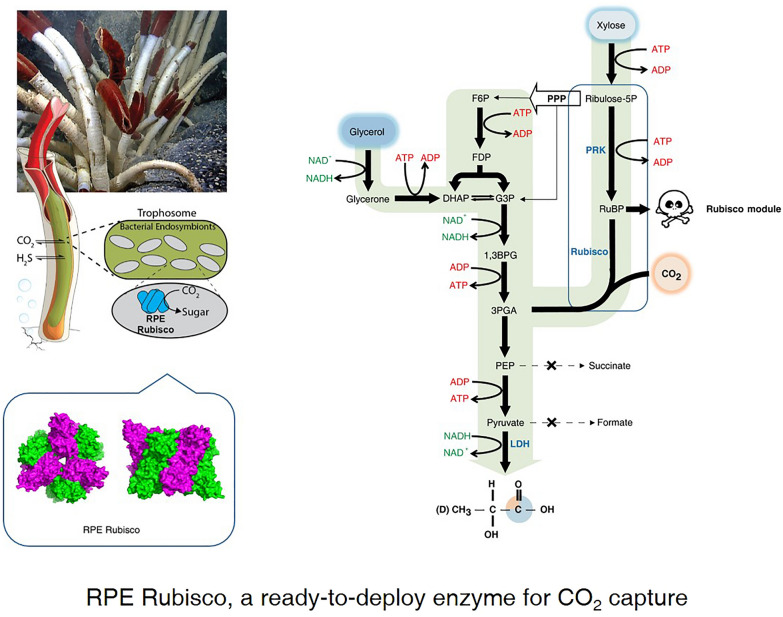

## Introduction

Photosynthesis uses light energy to convert inorganic CO_2_ into organic carbohydrates and forms the basis of most life on earth. Most of photoautotrophs employ ribulose-1,5-bisphosphate carboxylase/oxygenase (Rubisco) for CO_2_ fixation in the Calvin–Benson–Bassham (CBB) cycle. Extensive structural and biochemical studies have been performed on the Form I Rubisco enzymes from plants, algae and cyanobacteria. They share a similar hexadecameric structure composed of eight large subunits and eight small subunits, and are notorious for their low carboxylation activities, with turnover numbers toward CO_2_ in the range of 1–13 s^−1^ (Galmes et al. 2014; Hanson 2016; Whitney et al. 2011). Much effort in rational design and directed evolution has been made to improve their catalytic efficiencies, with limited success (Cai et al. 2014; Durao et al. 2015). Earlier studies by our group and others have demonstrated the feasibility of the Rubisco-based pathway for carbon fixation in *E. coli* (Gleizer et al. 2019; Gong et al. 2015; Zhuang et al. 2013), using phosphoribulokinase (PRK) and Form I Rubisco from cyanobacteria *Synechococcus*. Although cyanobacterial Rubisco exhibit the highest activity among the Form I Rubiscos, the difficulty to achieve efficient heterologous soluble expression in *E. coli*, and other hosts, hampers the carbon fixation efficiency (Orr et al. 2020; Wilson et al. 2018; Zhou and Whitney 2019).

**Fig. 1:**
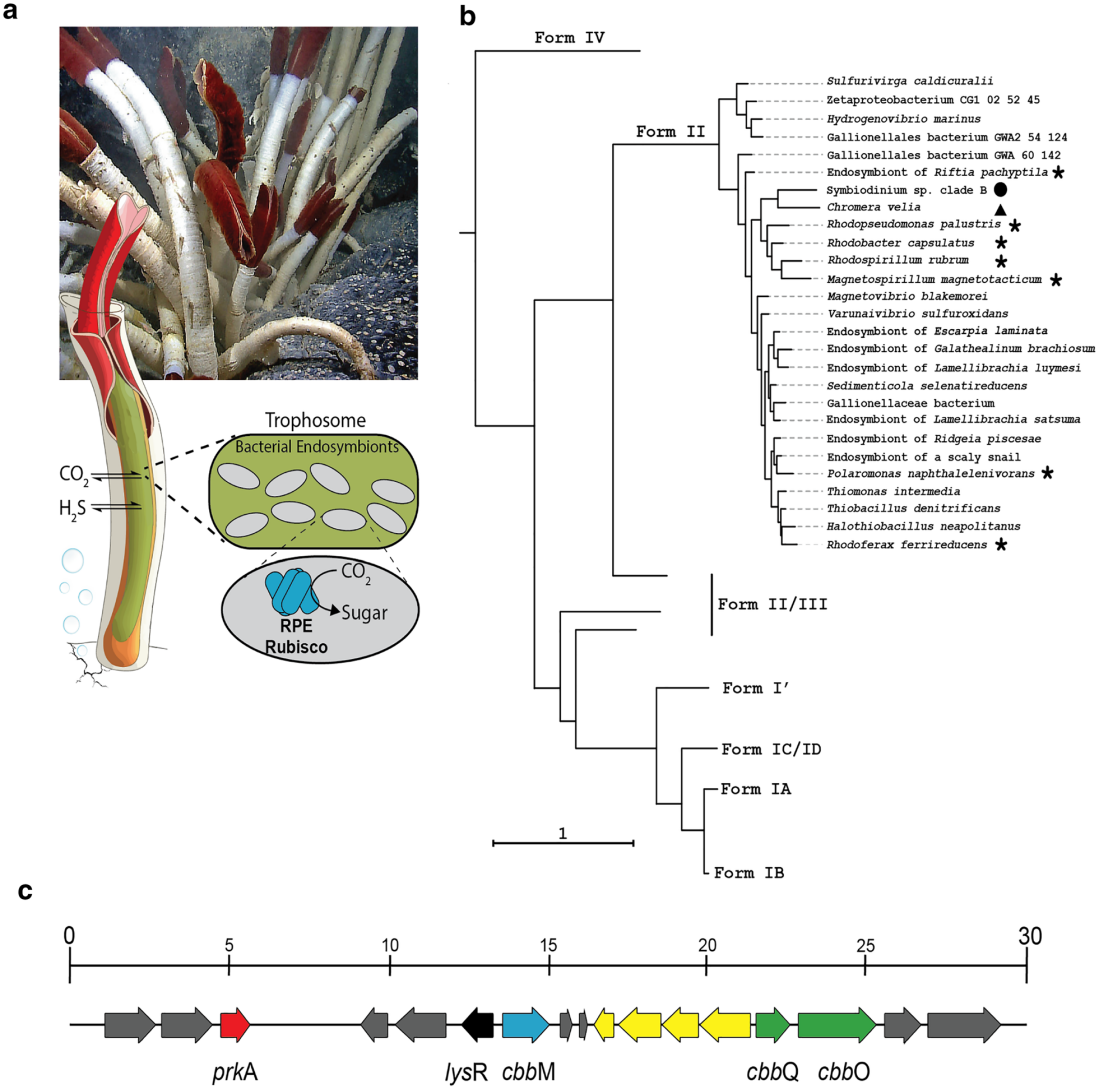
Chemosynthetic carbon fixation by the vestimentiferan tube worm *Riftia pachyptila*.** a** Deep sea vent environments provide a rich source of carbon dioxide (CO_2_) and hydrogen sulfide (H_2_S). Endosymbiotic chemoautotrophic bacteria in the trophosome organ of *R. pachyptila* use Rubisco to fix CO_2_ into sugar for the host using the reducing power of inorganic compounds such as H_2_S. *R. pachyptila* illustration adapted from (Hinzke et al. 2021). **b** Simplified phylogenetic tree of the Rubisco large subunit (RbcL) protein sequences. Endosymbiont Rubisco cluster in the Form II (RbcL_2-6_) clade of Rubisco. Dinoflagellate and Alveolate coral endosymbiont Rubiscos indicated by a filled circle and triangle, respectively. Species Rubiscos analyzed in this study are indicated with a star. Tree was rooted to Rubisco-like proteins (Form IV). Full phylogeny visible in Additional file 2: Figure S1. **c** *R. pachyptila* Rubisco operon and ~ 30 kbp local genomic neighborhood. Gene annotation: Red, phosphoribulokinase (prkA); black, Rubisco operon regulator (*lys*R); blue, Form II Rubisco large subunit (*cbb*M); yellow, glycolate oxidase gene cluster; green, Rubisco activase complex (*cbb*Q, *cbb*O); grey, unrelated/unidentified conserved genes

Nevertheless, nature has generated a variety of different Rubisco enzymes over more than 3.5 billion years of evolution (Fig. 1b) (Price et al. 2013). In addition to the extensively studied hexadecameric Form I Rubiscos, many Form II and Form III Rubiscos from anoxygenic photoautotrophic, chemoautotrophic or heterotrophic bacteria and archaea have also been identified (Tabita et al. 2008). These forms lack the small subunit and assemble as different hierarchies of large subunit dimers, which share low (~ 30%) primary sequence similarity to Form I Rubisco, with the exception of a recently characterized ancestral Form I’ clade which also lacks RbcS, but accordingly shows much higher similarity (~ 50%) to bona fide Form I members (Banda et al. 2020). To date, limited enzymological data were available regarding the catalytic parameters of these Rubisco enzymes. Most of the characterized Form II and Form III Rubiscos had far lower activities than that of the conventional Form I Rubisco from *Synechococcus* (Whitney et al. 2011) until a recent study reported a few highly active Form II Rubiscos (Davidi et al. 2020).

The aim of this study was to identify and characterize novel Rubisco with potential for carbon capture. To this end, seven Form II Rubiscos from a variety of microbes were selected for characterization of their carboxylation catalytic efficiency according to the diversities of classifications, growth types and habitats of their hosts. Heterologous expression and assembly level of these Rubiscos were evaluated. The high expression yields and activity of the endosymbiont Rubisco, particularly in comparison to cyanobacterial Rubisco, led us to investigate its structural properties. To demonstrate the potential application of RPE Rubisco, a bacterial carbon capture model was designed to characterize its CO_2_ fixation capability in vivo as a versatile tool for microbial CO_2_ capture platforms.

## Materials and method

### Plasmids construction

Seven Rubisco enzymes-encoding genes were selected and synthesized according to their NCBI accession numbers (Additional file 1: Additional Rubisco sequences information). All genes were inserted between the *Nde* I/*Xho* I sites of pET30a-7002-PRK (Additional file 2: Table S1) to replace the original 7002 Rubisco on the plasmid. A his_6_-tag was fused to the C terminus of the large subunit of the selected Rubisco enzymes for purification.

### Rubisco expression and purification

A single colony of the *E. coli* BL21(DE3) strain harboring Rubisco was inoculated into LB medium containing 50 ng μL^−1^ of kanamycin and cultured overnight at 37 °C. An aliquot comprising 200 μL of the overnight culture was inoculated into 20 mL of fresh LB medium containing 50 ng μL^−1^ of kanamycin. The culture was shaken at 37 °C until its OD_600_ reached 0.8–1. Then, IPTG was added to a final concentration of 0.05 mM. The temperature was reduced to 22 °C and Rubisco expression was continued for 6 h. Cells harvested from 2 mL of the culture were resuspended in 1 mL of Rubisco buffer A (50 mM HEPES, pH 8.0, 10 mM MgCl_2_, 5 mM KCl, 0.5 mM EDTA, 5 mM DTT). The cells were disrupted by ultrasonication and clarified by centrifugation to obtain the supernatant, which was cellular solution protein. An aliquot of 10 μL and 20 μL of cellular solution protein were subjected to SDS-PAGE (12%, w/v), and native-PAGE (6%, w/v). The his-tagged Rubisco in the cellular solution protein was purified using a nickel-chelating His∙Bind column (Novagen) and the buffer was exchanged for Rubisco buffer B (50 mM Tris–HCl, pH 8.0, 10 mM MgCl_2_, 1 mM EDTA) using YM-30 Microcon device (Millipore).

### Rubisco activity assay

Carboxylation activity of Rubisco in cellular solution protein was assayed using NaH^13^CO_3_ (Sigma) as substrate in an anaerobic chamber. Two hundreds microliter aliquots of crude cell extracts were activated for 30 min on ice with 20 μL of 200 mM NaH^13^CO_3_. The reaction was initiated by adding 13.5 μL of 29.6 mM d-ribulose-1,5-bisphosphate (RuBP, sigma) and conducted for 5 min at 25 °C. An aliquot comprising 60 μL of the reaction mixture was taken out and stopped by adding 20 μL of 1 M HCl. The solution was then neutralized with 20 μL of 1 M NaOH, and subjected to LC/MS/MS to determine the amount of ^13^C-3PGA produced (Gong et al. 2015). One unit of carboxylation activity was defined as the amount of enzyme that produces 1 μmol of ^13^C-3PGA per minute under the described condition.

Purified enzymes were subjected to the standard ^14^CO_2_ activity assay (Kubien et al. 2011; Wilson et al. 2018, 2019b) with modifications. Briefly, carboxylation activity of Rubisco was assayed anaerobically by preparing the reaction mixture in air-tight GC glass vials (Shimadzu) in an anaerobic chamber. The NaH^14^CO_3_ (PerkinElmer) and deoxygenated RuBP solution needed for initiating the reaction were added using air-tight glass syringes. Each vial consisted of 260 μL of Rubisco buffer B containing 30 mM NaHCO_3_, 2 μL of NaH^14^CO_3_, and 20 μL of purified enzyme. The reaction mixture was activated for 30 min at 25 °C followed by addition of 20 μL of 5 mM RuBP to start the reaction. The background control reactions were initiated by with Milli-Q ddH_2_O instead of RuBP. Each reaction was conducted at 25 °C for 2 min and stopped by the addition of 100 μL of 50% (v/v) formic acid. Reactions were dried in heat block at 80 °C, dissolved in 0.25 mL of H_2_O, mixed with 1 mL of Ultima-Gold scintillant (PerkinElmer), and measured for radioactivity in a scintillation counter (PerkinElmer).

The ^14^C-labeled 2-carboxyarabinitol-1,5-diphosphate (CABP) was synthesized by RuBP and ^14^C-KCN (American Radiolabeled Chemicals, Inc.) as before (Andersson et al. 1983; Pierce et al. 1980). Active sites of Rubisco in purified enzymes were determined as reported (Kubien et al. 2011). The ^14^CO_2_ carboxylation activity was defined as the molar amount of fixed ^14^CO_2_ divided by the molar amount of active sites determined by the ^14^C-labeled CABP binding per second. Rubisco soluble cellular concentration (%CSP) was obtained by dividing the Rubisco-soluble protein expressed by total cellular solution protein (Wilson et al. 2018, 2019b). Among them, Rubisco-soluble protein expressed was calculated by multiplying the nmol active sites according to the^14^C-CABP-binding by Rubisco molecular weight, the total cellular soluble protein was assayed by A_280_ using NanoDrop ND-1000. Furthermore, the enzyme activity (nmol CO_2_ fixed min^−1^ mg^−1^) was specified as the molar amount of fixed ^14^CO_2_ divided by total cellular solution protein, which CO_2_ fixation was assayed by ^14^C-method as mentioned above.

### Measurement of kinetic parameters

Kinetic parameters for CO_2_ of the purified enzymes were measured anaerobically using NaH^14^CO_3_ (Wilson et al. 2018). A reaction mixture consisted of 210 μL of Rubisco buffer B containing 10 μg/mL carbonic anhydrase and 30–1800 μM CO_2_ and 20 μL of 10 mM RuBP was prepared in air-tight GC glass vials in an anaerobic chamber. NaH^14^CO_3_ was added by air-tight glass syringes. The assays were initiated by the addition of 20 μL of preactivated Rubisco by gas-tight syringes, incubated at 25 °C for 2 min, and stopped by 100 μL of 50% (v/v) formic acid. Determination of radioactivity and active sites were the same as above. Kinetic parameters were calculated by non-linear fitting to the Michaelis–Menten equation by software OriginPro 8.5.

### Crystallization and structure determination

Purified RPE Rubisco was buffer-exchanged into a solution containing 10 mM Tris–HCl (pH 8.0) and 100 mM NaCl. The protein concentration was determined to be 10 mg mL^−1^. Seven 96-well crystal screening kits were set up for crystallization using the sitting drop vapor diffusion method by mixing equal volumes of protein and a buffer solution containing 1.26 M sodium phosphate monobasic monohydrate and 0.14 M dibasic potassium phosphate at 18 °C. The best diffracting apo crystals were grown using reservoir solution comprising 0.15 M magnesium formate and 12% (w/v) polyethylene glycol 3350. Crystals were rapidly soaked in reservoir solution supplemented with 20% glycerol as cryoprotectant, mounted on loops, and flash-cooled at 100 K in a nitrogen gas cryo-stream. Crystals diffraction data were collected from a single crystal at the BL18U beamline of the Shanghai Synchrotron Radiation Facility (SSRF, China), with a wavelength of 0.9793 Å at 100 K. The diffraction data were processed and scaled using HKL-3000 (Otwinowski and Minor 1997). The relevant statistics are summarized in Additional file 2: Table S2. The structure was solved by the molecular replacement method using *Gallionellacea* Rubisco (PDB code 5C2C as the starting model. The initial model was built using PHENIX autobuild (Adams et al. 2002). Manual adjustment of the model was carried out using the program COOT (Emsley and Cowtan 2004) and the models were refined using PHENIX refinement (Adams et al. 2002) and REFMAC5 (Murshudov et al. 1997). The stereochemical quality of the structures was checked using PROCHECK (Laskowski et al. 1993). All residues were found to be located in the favored and allowed regions and none in the disallowed regions. Refinement resulted in a model with excellent refinement statistics and geometry (Additional file 2: Table S2). The coordinates of RPE Rubisco have been deposited in Protein Data Bank under the PDB code 6IUS.

### Cell growth and d-lactate production using Rubisco CO_2_ fixation

For comparing cell colonies growth on the plate, 1 mL overnight cell culture in LB broth with 50 ng μL^−1^ of kanamycin was harvested by centrifugation (10,000 g, 1 min), washed and resuspended in 0.9% NaCl twice, followed by gradient dilution, 10 μL of a 10^–3^,10^–4^, 10^–5^, 10^–6^ dilution was individually spotted on M9 minimum medium (5 g L^−1^ xylose, 6.13 g L^−1^ glycerol, 0.5 g L^−1^ casamino acid, 0.05 mM IPTG, 50 ng μL^−1^ of kanamycin) and cultured in 10% CO_2_, 90% air, 25 °C. For comparing cell growth in liquid medium, 1:100 (1%, v/v) overnight culture overnight cell culture in LB broth with 50 ng μL^−1^ of kanamycin was inoculated into 50 ml fresh M9 minimum medium and cultured under the same condition as above.

After Rubisco expression in LB broth, 10 mL cell culture was harvested and washed by 0.9% NaCl, cell supernatants were collected at an OD_600_ of 0.20 ± 0.02, resuspended in 10 ml M9 minimal medium in addition with 100 mM HEPES and 100 mM NaH^13^CO_3_, pH 7.0. Following incubation for 3 days in anaerobic condition at 30 °C and regular sampling, cell growth was monitored using a 96-plate reader (Infinite 200pro, Tecan). The consumption of xylose and glycerol in the supernatant was determined using Agilent 1200 Infinity HPLC system with an Aminex HPX-87H column (300*7.8 mm, Bio-Rad) and refractive index detector. Samples were run at 55 °C and eluted at 0.6 ml/min with 5 mM sulfuric acid. The d-lactate production including the ^13^C-labeled d-lactate concentration was carried out by QTRAP 6500 LC–MS/MS with Multiple Reaction Monitoring (MRM) mode. The temperature of electron spray ionization (ESI) was 550 °C, and the detection voltage was -4500 V. Samples were analyzed with a 10-µL injection onto a HyperREZ XP column (7.7*100 mm) heated to 40 °C. The deionized water eluted for 10 min at a flow rate of 0.4 mL min^−1^.

### Metabolic flux analysis

The constrained metabolic flux was analyzed according to the Rubisco-based CO_2_ capture model. The consumption of xylose (Xyl_total_) was separated into pentose phosphate pathway (X_1_) and Rubisco bypass pathway (X_2_), which was calculated by Eq. 1. The mole of F6P was calculated 5/6 X_1_ by carbon rearrangement though pentose phosphate pathway. The generated G3P (X_3_) was derived from F6P and glycerol metabolism (Gly_total_), as shown in Eq. 2:

1 $${\text{Xyl}}_{{{\text{total}}}} = \, X_{{1}} + \, X_{{2}} ,$$

2 $$X_{{3}} = { 2} \times \frac{5}{6}X_{{1}} + {\text{ Gly}}_{{{\text{total}}}} .$$

The important intermediate metabolite 3PGA was produced by central carbon metabolism and Rubisco-based CO_2_ capture bypass pathway, and the mole of 3PGA was the summation of the mole of G3P and the mole of uptake CO_2_ (*X*_2_). Under this premise, that NADH production was greater than or equal to NADH consumption, it was assumed that ^13^C-labeled ratio of 3PGA (R_3PGA_) was equal to the ratio of the detected labeled lactate, so the detected ratio of ^13^C-labeled lactate (R_lactate_, after deducting the background interference) was calculated by X_2_ and X_3_ in Eq. 3.

As NADH production ≥ NADH consumption,

3 $$R_{{{\text{3PGA}}}} \% \, = \frac{{X_{2} }}{{X_{3} + 2X_{2} }} = R_{{{\text{lactate}}}} \% .$$

The ^13^C-labeled d-lactate was detected at the end of 72 h, the consumption rates and the production rates, and the uptake CO_2_ flux were calculated in the last 12 h.

### Rubisco large-subunit protein phylogenetic analysis

Eighty six Rubiscos were manually selected to have representatives of the different groups of Rubisco, focusing specially on Type II Rubiscos and Rubiscos reported from endosymbionts. This set of Rubisco sequences was then aligned and trimmed using MAFFT (Katoh et al. 2002) in Guidance2 server (Sela et al. 2015). The columns with scores lower than 0.93 were removed and subsequently, a maximum-likelihood phylogenetic was built using PhyML 3.0 (Guindon and Gascuel 2003) with 1000 Bootstraps and a LG model for amino acid substitution.

### Statistical analysis

The statistical analysis of data and plots was performed using an unpaired 2-tailed Student’s t test in GraphPad Prism software version 7.0. *P* values of < 0.05 were considered to indicate statistical significance. Data are presented as means ± standard errors of the mean.

## Results

### Discovery of a highly active Form II RPE Rubisco

Seven Form II Rubiscos from different microbes were selected for characterization (Additional file 2: Table S1). These 7 microbes belong to 3 classes, 4 orders, 4 families, and 7 genera, and are capable of photoautotrophic, chemolithoautotrophic, and heterotrophic growth. They live in diverse environments including extreme habitats such as deep-sea hydrothermal vents and cold lakes. The multiple sequence alignment was provided and the similarity of each two Rubiscos amino acid sequences was about 79% ~ 91%. Their Rubisco genetic evolutionary relationship was analyzed by the simplified phylogenetic tree (Fig. 1b).

The large subunit gene of each selected Rubisco (RbcL) was placed under the control of a T7 promoter in a pET30a plasmid and expressed in *E. coli* BL21(DE3). A representative cyanobacterial Rubisco from *Synechococcus* sp. PCC 7002 (7002 Rubisco) was used as a reference for Form I Rubisco expression yield and activity. The large and small subunit genes of 7002 Rubisco, together with *rbc*X which encodes a Rubisco specific assembly chaperone that improves enzyme yield in *Escherichia coli* (Emlyn-Jones et al. 2006), were cloned into pET30a from our previous study (Cai et al. 2014). All the selected Form II Rubiscos were expressed and assembled in *E. coli* BL21(DE3) without addition of any foreign chaperones (Additional file 2: Figure S2a, b). For most of these Form II Rubiscos, the expression/assembly levels and the carboxylation activities assessed using cellular solution protein were higher than those of 7002 Rubisco expressed with the assistance of RbcX. Among them, the Rubisco from endosymbiotic bacteria which lived in the trophosome of the *Riftia pachyptila* (RPE Rubisco) showed the highest carboxylation activity in cellular solution protein (Additional file 2: Figure S2c). RPE Rubisco could be the key enzyme of ubiquitous chemosynthetic symbioses between invertebrate and bacteria to fix CO_2_ using H_2_S as energy to support the rapid growth of the host (Fig. 1a) (Hinzke et al. 2021; Li et al. 2018). Interestingly, RPE Rubisco resides in a separate clade away from other endosymbionts (Fig. 1b). Its operon and ~ 30-kbp local genomic neighborhood contains coding genes of Rubisco (*cbb*M), phosphoribulokinase (*prk*A), and Rubisco activase complex (*cbb*Q, *cbb*O) (Fig. 1c).

### Kinetic parameters and solubility of RPE Rubisco

Both RPE and 7002 Rubiscos were purified by nickel affinity chromatography after fusion with a his_6_-tag at the C terminus of the large subunit. Standard ^14^CO_2_ activity assay revealed a *k*_cat_^C^ value for RPE Rubisco of 16.4 s^−1^ at 25 °C, whereas 12.3 s^−1^ for 7002 Rubisco. The *K*_M_^C^ value of RPE Rubisco was 11% lower compared to 7002 Rubisco. The resulting carboxylation efficiency (*k*_cat_^C^/*K*_M_^C^) of RPE Rubisco was thus 50.5% higher than that of 7002 Rubisco (Table 1).

**Table 1:** Kinetic parameters of 7002 and RPE Rubiscos

Enzyme	*k*_cat_^C^ (s^−1^)	*K*_M_^C^ (μM)	*k*_cat_^C^/*K*_M_^C^ (s^−1^ mM^−1^)
7002 Rubisco	12.3 ± 0.7	194.6 ± 35.7	63.2 ± 8.0
RPE Rubisco	16.4 ± 0.7	172.4 ± 23.6	95.1 ± 9.0*

Data represent mean ± standard deviation of at least three independent measurements. *P*-values relative to 7002 Rubisco indicated significance at *P < 0.05, **P < 0.01

**Fig. 2:**
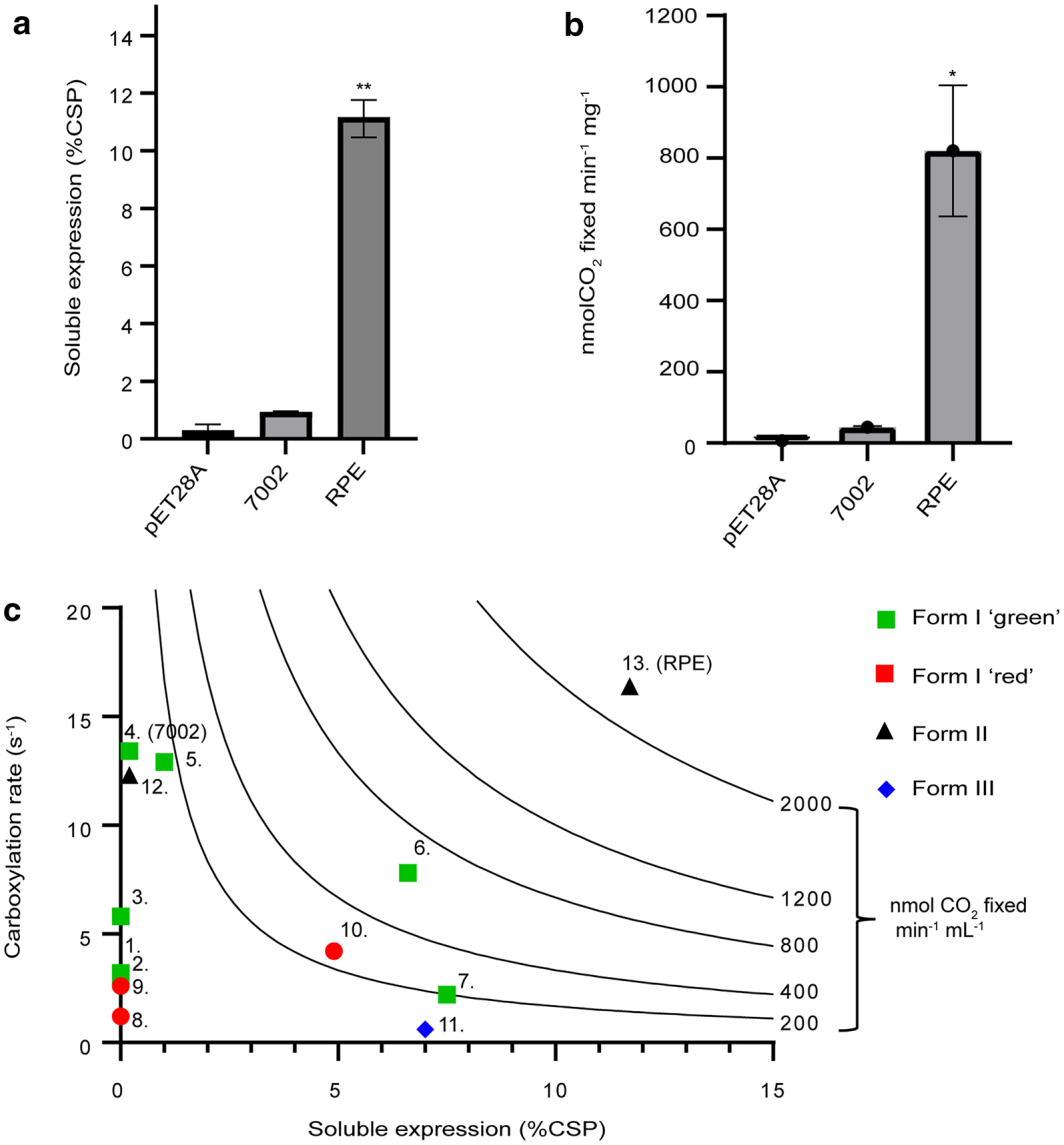
Comparative analysis of carboxylation activity and solubility between 7002 and RPE Rubisco by ^14^C-labled method.** a** Rubisco soluble cellular concentration as a percentage of total cellular solution protein (%CSP), **b** The enzyme activity specified as the molar amount of fixed ^14^CO_2_ divided by total cellular solution protein, **c** Rubisco maximal carboxylation rates (*V*_max_) and soluble expression yields in *E. coli* as reported %CSP and according to labeling in Additional file 2: Table S3. The %CSP values are represented as the observed or reported yield when no additional chaperones are co-expressed alongside the Rubisco genes. Contour lines represent the %CSP yield and Rubisco carboxylation rate (*V*_max_) required to achieve a maximum theoretical CO_2_ fixation rate in vivo. This rate is represented in nmol CO_2_ fixed min^−1^ mL^−1^ for a bioreactor culture that has an assumed average protein concentration of 1 mg mL^−1^. *P*-values relative to 7002 strain indicated significance at *P < 0.05, **P < 0.01

In addition to the higher carboxylation efficiency, the solubility of RPE Rubisco in *E. coli* was also noteworthy. Based on the active sites of Rubisco that were determined by ^14^C-labeled CABP binding (Kubien et al. 2011), the cellular concentration of RPE Rubisco was calculated to be 12% of the cellular solution protein (%CSP), which is about 12-fold higher than 7002 Rubisco (Fig. 2a). Such a high cellular concentration of RPE Rubisco coincides well with its high-level soluble expression and assembly. Consequently, the in vitro CO_2_ fixation rate of RPE Rubisco in bacterial crude extracts reached up to 820 ± 182 nmol CO_2_/min^−1^/mg CSP, which was 19-fold higher than 7002 Rubisco (Fig. 2b), and eightfold higher in comparison to abundantly expressing higher plant (*Arabidopsis thaliana*) Rubisco in *E. coli* (Wilson et al. 2019b). In fact, most Form I Rubisco, with the exception of cyanobacterial isoforms like 7002 Rubisco, display carboxylation rate values (*k*_cat_^C^) that fundamentally limit host CO_2_ fixation rates at normal recombinant expression yields (Fig. 2c). As shown in this study and others (Lin et al. 2014; Orr et al. 2020; Wilson et al. 2018), the poor heterologous expression of cyanobacterial Rubisco provides an incredibly large barrier to effective CO_2_ capture utility outside of their normal hosts and in the absence of the CO_2_ concentrating apparatus and chaperones that support their function. In these terms, RPE Rubisco stands in a class of its own with the potential to capture carbon at a significant rate under elevated atmospheric CO_2_ concentrations and straightforward heterologous expression (Fig. 2c).

### Structure determination and analysis

**Fig. 3:**
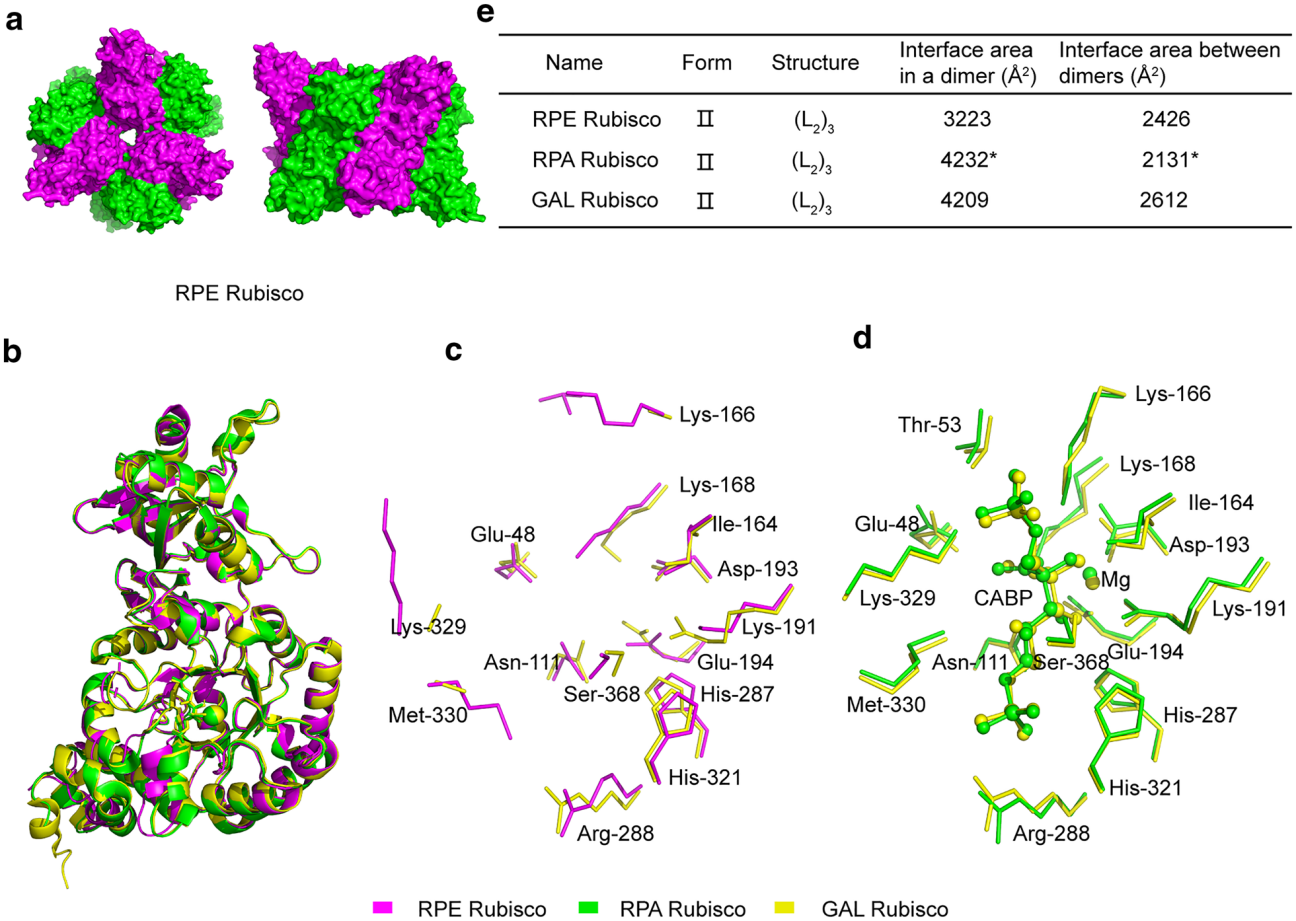
Structure of RPE Rubisco and its comparison with another two hexameric Form II Rubiscos. **a** Top and side views of RPE Rubisco (PDB code 6IUS). **b.** Superimposition of the large-subunit monomers of RPE (magenta), RPA (green, PDB code 4LF1), and GAL (yellow, PDB code 5C2G) Rubiscos. **c** Superimposition of active-site residues in RPE (PDB code 6IUS) and GAL Rubiscos (PDB 5C2C) without ligand. **d** Superimposition of active-site residues in RPA (PDB code 4LF1) and GAL Rubiscos (PDB code 5C2G) with ligand CABP. The residues and CABP were shown in sticks and spheres, respectively. The residues were numbered according to GAL Rubisco. **e** Comparison of interface areas within a dimer and between two dimers (Å^2^) of the three hexameric Form II Rubiscos, the symbol of ‘*’ noted the data from Satagopan, et al. (2014)

Analytical ultracentrifugation revealed that RPE Rubisco has a molecular weight of 322 kDa (Additional file 2: Figure S3), indicating that it is a hexamer. Structure resolution confirmed this and showed that RPE Rubisco (PDB code 6IUS) consisted of three pairs of homodimers of the large subunit arranged around a central threefold symmetry axis (Fig. 3a). Structural comparison of RPE Rubisco and two previously reported hexameric RPA and GAL Rubisco (PDB codes 4LF1 and 5C2G, respectively) (Satagopan et al. 2014; Varaljay et al. 2016) showed a strong similarity, which can be ascribed to their high identity of amino acid sequences (73–78%). Superimposition of their monomeric large subunits revealed that the Cα backbones were virtually identical (Fig. 3b). Residues within 4 Å of CABP were conserved among the three Rubiscos. Fifteen active-site amino acids predicted based on the protein sequence of Rubisco are labeled in Fig. 3c, d. The active-site geometries of RPE and GAL Rubiscos without ligands are highly similar, showing the “apo” forms (Fig. 3c). The “activated” forms of RPA and GAL Rubiscos with CABP binding were also highly similar (Fig. 3d). The main conformational changes between the “apo” and “activated” forms occurred in residues Lys 166, Met 330, and Lys 329 (Fig. 3c). These three active-site amino acids were potentially related with the major conformational changes accompanying the reaction of Rubisco with substrate RuBP. Lys 166 and Lys329 participate in catalysis (Cleland et al. 1998), thus cannot be substituted. Met 330 residue described above in RPE Rubisco is similar to that of Met 331 in RPA Rubisco loop 6, which closes an active site in conjunction with residues from a neighboring subunit of homodimer. The *K*_cat_ of mutant enzyme M331L was decreased about 95% (Satagopan et al. 2014), highlighting the importance of Met 331 in RPA Rubsico. Likewise, Met 330 might also play an indispensable role in RPE Rubisco. Slight differences were observed when comparing their oligomeric structures. The interaction surfaces within one dimer and between two neighboring dimers of RPE Rubisco were 3223 A^2^ and 2426 A^2^, respectively, while those for RPA Rubisco were 4232 A^2^ and 2131 A^2^, and 4209 A^2^ and 2612 A^2^ for GAL Rubisco (Fig. 3e).

### Creation of a Rubisco-based CO_2_ capture model

**Fig. 4:**
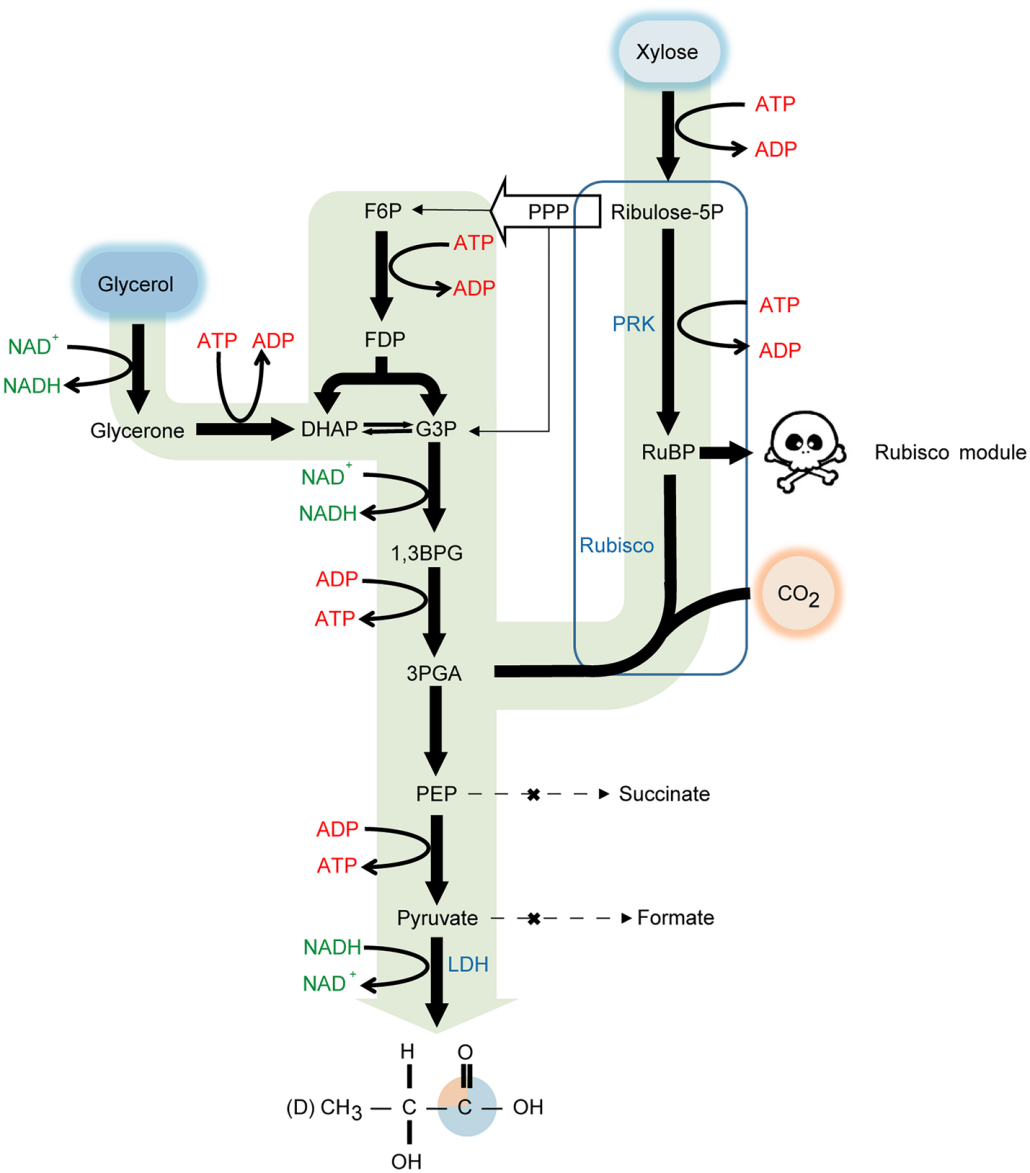
Schematic diagram of Rubisco carbon capture to produce d-lactate in *E. coli*. PRK, phosphoribulokinase, Rubisco, ribulose-1,5-bisphosphate carboxylase/ oxygenase, LDH, Lactate dehydrogenase, RuBP, ribulose 1,5-bisphosphate, 3PGA, 3-phosphoglycerate, PEP, phosphoenolpyruvate. “X” on the dotted arrows indicates that metabolic pathway was blocked. The skull and crossbones on the right side of RuBP indicates the toxicity induced by accumulation of RuBP. Orange quadrant of carboxyl carbon of the d-lactate indicates the exact position where the carbon of CO_2_ is captured, and one out of four d-lactate produced, theoretically, would have captured carbon in that position

To investigate whether the higher carboxylation catalytic efficiency or heterologous expression of RPE Rubisco could improve heterotrophic CO_2_ fixation in *E. coli*, a CO_2_ capture model was designed. The model should be able to evaluate the activity of different Rubiscos and the efficiency of CO_2_ capture. To this end, the basic idea was to choose xylose as the starting substrate to capture CO_2_ through Rubisco. Moreover, the xylose and the captured CO_2_ are directed towards the production of d-lactate so that CO_2_ is deposited in the form of d-lactate (Fig. 4). Three principles were considered when developing this model.

First, since xylose can be metabolized by *E. coli* through the pentose phosphate pathway, selective pressure must be introduced to force xylose to be used to capture CO_2_. PRK and Rubisco were thus cloned under an inducible promoter and transformed into the host cell. It is known that d-ribulose-1,5-bisphosphate (RuBP), the product of PRK-catalyzed reaction, is toxic to *E. coli*. The growth of cells containing active PRK will thus be retarded or even repressed (Cai et al. 2014; Hudson et al. 1992; Mueller-Cajar et al. 2007). Rubisco converts RuBP into glycerate-3-phosphate (3PGA), an intermediate of glycolysis. The higher the activity of Rubisco, the quicker the growth inhibition can be relieved via catalytic processing of the dead-end metabolite RuBP in these cells. By comparing the resulting growth profile, the relative carboxylation activity of different Rubiscos in vivo can be assessed.

Second, pyruvate is the end product of glycolysis, but since pyruvate can be easily channeled into different metabolism pathways, d-lactate was chosen as the end product for CO_2_ capture. To maximize the carbon flux towards d-lactate, a gene *ldh* encoding d-lactate dehydrogenase from *Lactobacillus delbrueckii* was expressed, while the gene *pflB* encoding pyruvate formate-lyase and the genes *frdABCD* encoding succinate dehydrogenase were inactivated to block the conversion of pyruvate to acetyl-CoA and succinate, respectively.

Third, it is expected that under ideal anaerobic conditions no CO_2_ will be released once captured. Under such a condition, the NADH generated in association with xylose catabolism from xylose is insufficient if xylose is used as the sole substrate. Thus, additional NADH must be provided as shown in formula (1). The shortage of NADH can be provided by glycerol metabolism as shown in formula (2). Consequently, by using glycerol as a co-substrate, a general formula (3) could be deduced. In this conversion process, captured CO_2_ will accumulate in the form of d-lactate. The amount of labeled fraction of CO_2_ in d-lactate can be used to assess the efficiency of labeled CO_2_ capture.

1 $${\text{Xylose }} + {\text{ 2 NADH }} + {\text{ CO}}_{{2}} \to {\text{ 2 Lactate }} + {\text{ 2 NAD}}^{ + }$$

2 $${\text{Glycerol }} + {\text{ ADP }} + {\text{ NAD}}^{ + } \to {\text{ Lactate }} + {\text{ ATP }} + {\text{ NADH}}$$

3 $${\text{Xylose }} + {\text{ 2 Glycerol }} + {\text{ CO}}_{{2}} + {\text{ 2ADP }} \to {\text{ 4 Lactate }} + {\text{ 2 ATP}}$$

### Characterization of Rubisco-based CO_2_ capture in vivo

**Fig. 5:**
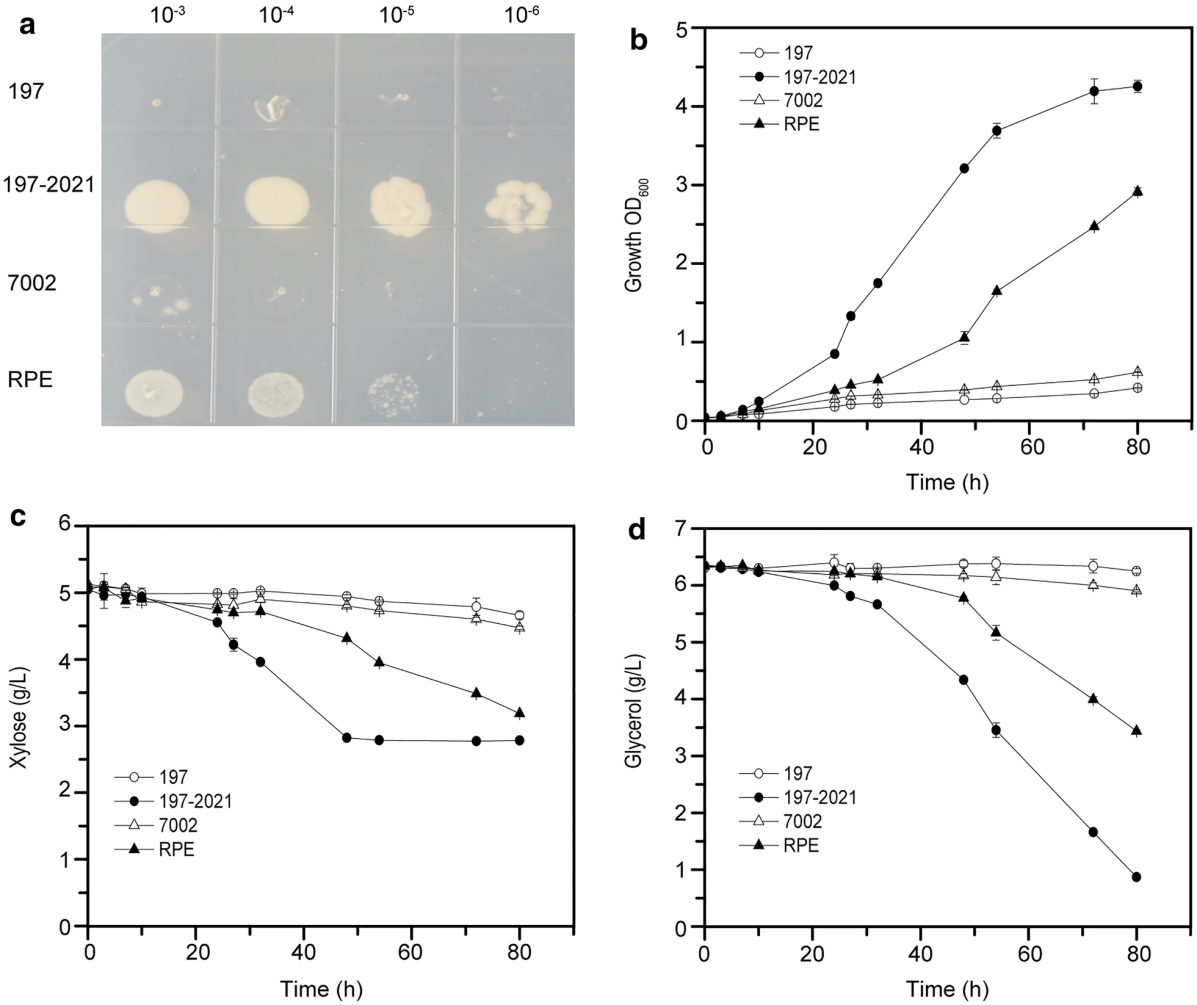
Growth profile of strains expressed by different Rubiscos. 197, negative control strain with active PRK but inactivated Rubisco; 197-2021, positive control strain with inactivated PRK and Rubisco; 7002, strain with PRK and 7002 Rubisco; RPE, strain with PRK and RPE Rubisco (details can be found in Additional file 2: Table S1). **a** Cell cultures were spotted on M9 minimal medium plate and incubated in 10% CO_2_, 90% air. **b** Cell growth of strains in M9 minimal medium under the same conditions as above. **c**, **d** Xylose and glycerol consumption of strains under the same conditions as above

The model strain constructed following the above principles was designated as BWLac (BW25113Δ*frd*ABCDΔ*pfl*B::*ldh*A). BWLac strains with plasmids pET-RBC197-PRK, pET-RBC197-PRK2021, pET-7002-PRK and pET-RPE-PRK (Additional file 2: Table S1) were designated as strains 197, 197-2021, 7002 and RPE, respectively. Strain 197 contains an inactive 7002 Rubisco as a K197M mutation was introduced into the conserved catalytic site of Rubisco large subunit (Cai et al. 2014). Strain 197-2021 contains an inactive 7002 Rubisco (K197M) and an inactive PRK where K20M/S21A mutations were introduced into the conserved nucleotide binding site of ATP-binding proteins (Cai et al. 2014; Higgins et al. 1986; Wilson et al. 2019a). According to the Rubisco-based CO_2_ capture model, strain 197 containing an active PRK but inactive Rubisco was crippled by RuBP toxicity leading to drastic growth defects and therefore is used as a negative control for relief by Rubisco. Strain 197-2021 contains both inactive PRK and inactive Rubisco leading to a near wild-type growth rate, which is used as control (Fig. 5a, b). Strains expressing RPE Rubisco grew much faster than strains utilizing 7002 Rubisco, in either plate or liquid culture (Fig. 5a, b). Compared with strain 7002 and the negative control strain 197, strain RPE was able to rapidly grow and metabolize both xylose and glycerol. The growth profiles of the four tested strains on agar plates (Fig. 5a) and in shake flasks (Fig. 5b) were consistent. This suggests that the RPE Rubisco quite effectively released the toxicity caused by the accumulation of RuBP. Interestingly, the positive control strain 197-2021, which showed rapid growth and achieved the highest biomass, did not consume xylose once the residual xylose concentration reached less than 2.8 g/L after 48 h, while glycerol could be continuously utilized (Fig. 5c, d). This suggests the overall activity of RPE Rubisco in strain RPE is very high, conferring the cell the capability to rapidly detoxify the RuBP generated by PRK and is consistent with the high activity of RPE Rubisco observed from cellular solution protein (Fig. 2b).

To quantitatively evaluate the CO_2_ capture rate, BWLac strains harboring different plasmids were incubated aerobically to first induce protein expression, then subjected to catalyze CO_2_ capture. In principle, d-lactate can also be produced by xylose metabolism through pentose phosphate pathway without Rubisco module. This means d-lactate can be produced by the positive control strain. Once the Rubisco module is introduced, the higher the carboxylation activity of Rubisco, the higher the production rate of lactate, and the more ^13^C labeled d-lactate from ^13^CO_2_ assimilation can be detected (Fig. 4). Thus, the titer and ratio of ^13^C-labeled lactate could be used to evaluate the efficiency of CO_2_ capture.

**Fig. 6:**
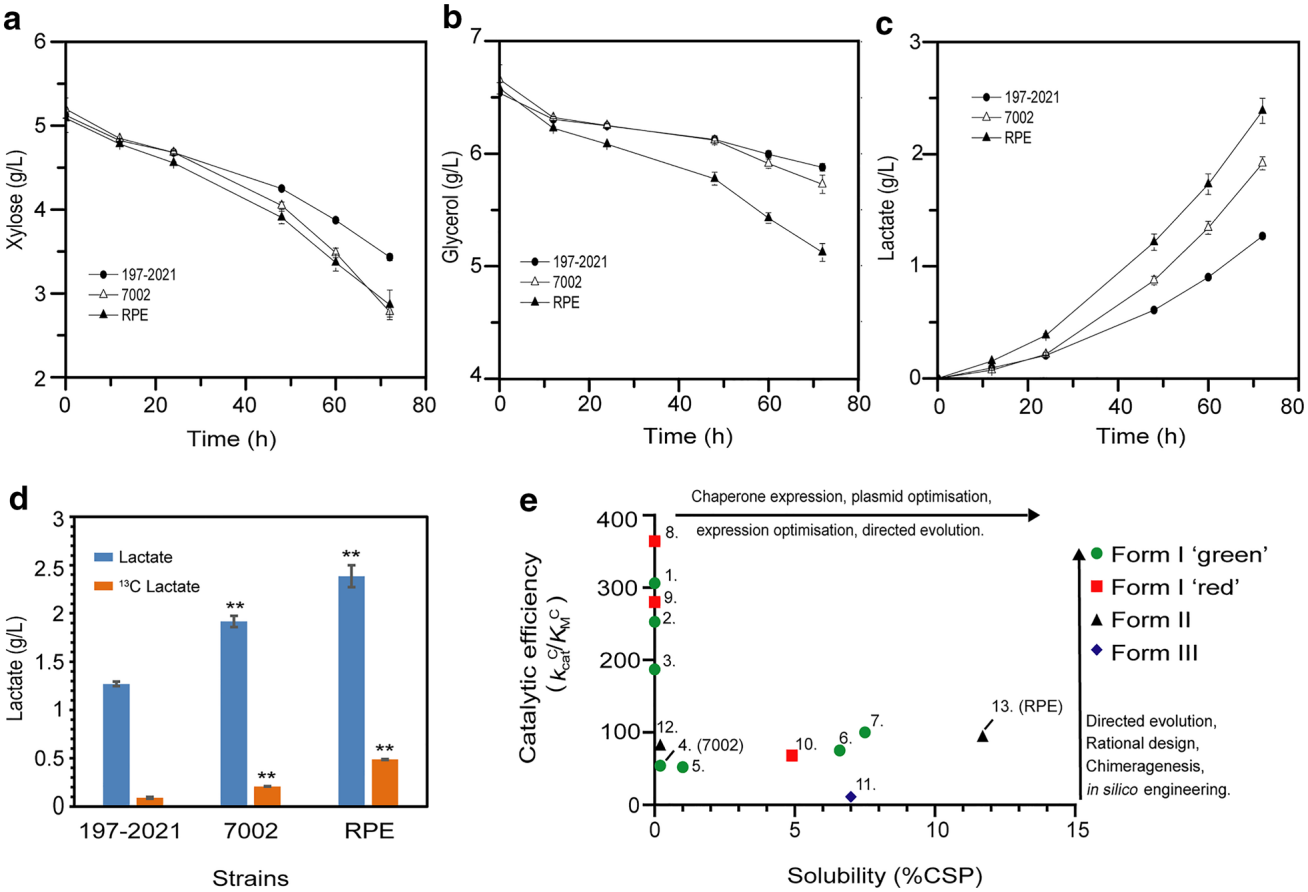
Characterization of Rubisco-based CO_2_ fixation ability in vivo. 197-2021, positive control strain with inactivated PRK and Rubisco; 7002, strain with PRK and 7002 Rubisco; RPE, strain with PRK and RPE Rubisco. Xylose, glycerol consumption **a**, **b** and d-lactate production **c**, **d** as methods described. The mean values and standard derivations of three independent repetitions were shown. *P*-values relative to 197–2021 indicated significance at *P < 0.05, **P < 0.01. **e** Plot of Rubisco carboxylation efficiency against heterologous solubility for the enzymes listed in Additional file 2: Table S3. For an organism expressing Rubisco for CO_2_ capture under anoxic conditions (no oxygen competition) optimality corresponds to the upper right region of the plot. Relevant engineering strategies for improving in vivo CO_2_ sequestration rates for different Rubisco enzymes are provided as a guide

In the carbon fixation experiment using the CO_2_ capture model that we constructed, the consumption of xylose and glycerol as well as the production of lactate of the positive control strain 197–2021 were the least among the three strains tested. The time-profile of strain 197–2021 serves as a background to calculate how much additional CO_2_ can be fixed through Rubiscos in strains RPE and 7002. Interestingly, the xylose consumption of strain RPE was similar to that of strain 7002, but the glycerol consumption of strain RPE was much faster (Fig. 6a, b). As a consequence, the lactate production of strain RPE was the fastest and the highest, followed by strain 7002 and strain 197-2021 (Fig. 6c). The ^13^C-labeled lactate concentrations from the fermentation broth collected at 72 h were analyzed, which were 0.09 ± 0.01 g/L, 0.21 ± 0.001 g/L, and 0.49 ± 0.003 g/L for strain 197–2021, strain 7002, and strain RPE, respectively (Fig. 6d). It is conceivable that the lower ^13^C-labeled lactate titer of strain 7002 was due to the lower carboxylation activity of 7002 Rubisco resulting from its poor heterologous expression/assembly in *E. coli*, which could also be reflected in its slow glycerol consumption (Fig. 6b and d). Overall, these results indicate that the Rubisco-based CO_2_ capture model functions as designed.

In addition to the production of d-lactate, by-products acetate and a small amount of ethanol were detected, but no formate was detected because of the knockout of gene *pfl*B. The carbon recovery of strain PRE reached up to 0.92, higher than that of strain 7002 and control strain 197–2021 (Table 2). As the carbon released from this process could not be quantified precisely, it is reasonable that none of the strains could achieve 100% carbon recovery. We also calculated the NADH/NAD^+^ production/consumption based on the carbon flux data from Table 2. The net flux of NADH in strain RPE is higher than that of strain 7002 and the control strain 197–2021 (Table 2), which is conceivable as the carbon fixation in strain RPE requires more NADH.

**Table 2:** Substrates consumption rates, products formation rates, carbon recovery and net flux of NADH in Rubisco-based CO_2_ capture model

	Substrates consumption rates and products formation rates^a^ (mmol gDCW^−1^ h^−1^)			Carbon recovery (*C* atoms)		
	197-2021	7002	RPE	197-2021	7002	RPE
Xylose	0.41 ± 0.01	0.67 ± 0.03	0.50 ± 0.11	2.05 ± 0.05	3.35 ± 0.15	2.50 ± 0.55
Glycerol	0.18 ± 0.03	0.28 ± 0.06	0.46 ± 0.07	0.54 ± 0.09	0.84 ± 0.18	1.38 ± 0.21
Uptake ^13^CO_2_^b^	–	0.05 ± 0.002	0.18 ± 0.04	–	0.05 ± 0.002	0.18 ± 0.04
Lactate	0.57 ± 0.02	0.89 ± 0.03	1.01 ± 0.11	1.71 ± 0.06	2.67 ± 0.09	3.03 ± 0.33
Acetate	0.11 ± 0.01	0.11 ± 0.02	0.18 ± 0.02	0.22 ± 0.02	0.22 ± 0.04	0.36 ± 0.04
Ethanol	0.01 ± 0.002	0.06 ± 0.01	0.06 ± 0.01	0.02 ± 0.004	0.12 ± 0.02	0.12 ± 0.02
Released CO_2_^c^				0.12 ± 0.01	0.17 ± 0.03	0.24 ± 0.03
Total carbon consumption				2.59 ± 0.14	4.24 ± 0.33	4.06 ± 0.80
Total carbon production				2.07 ± 0.10	3.18 ± 0.18	3.75 ± 0.42
Carbon balance				0.80 ± 0.01	0.75 ± 0.02	0.92 ± 0.08
Net flux of NADH^d^				0.56 ± 0.01	0.60 ± 0.03	0.74 ± 0.08

^a^Calculated by the detected molar concentration divided by dry cell weight, one unit OD_600_ = 0.3 gDCW/L (Soini et al. 2008), the cell density of samples remained stable around the initial 2 unit OD_600_

^b^The value of X_2_ was calculated by Eqs. 1, 2, 3 based on ^13^C-labeled lactate production

^c^Assumed that released CO_2_ was equal to the summation of the moles of acetate and ethanol in the dissimilation of pyruvate

^d^Calculated by the ratio of NADH consumption to NADH production, NADH consumption accompanied the production of ethanol and lactic acid, NADH production was from the reaction of glycerol to glycerone and G3P to 1,3BPG, one molecule of NADH consumed or produced per reaction

## Discussion

We report a highly active Form II Rubisco (RPE) from the endosymbiont of the deep-sea tubeworm *Riftia pachyptila*, which shows a 50.5% higher carboxylation efficiency (*k*_cat_^C^/*K*_M_^C^) than that of the high-performance Form I Rubisco from *Synechococcus* sp*.* 7002. Crucially, RPE Rubisco expresses to high levels in *E. coli* (12% CSP) without additional chaperones, even compared to other Form II enzymes which generally express well in heterologous hosts (Davidi et al. 2020; Whitney and Andrews 2003). Many studies have reported the active expression of cyanobacterial Form I Rubisco in *E. coli* and other hosts (Lin et al. 2014; Occhialini et al. 2016; Wilson et al. 2018) has long been regarded as the pinnacle of carboxylation efficiency for Rubisco (Davidi et al. 2020). The soluble expression of 7002 Rubisco in *E. coli* in our work was only about 1% CSP, even in the presence of chaperones to boost yield (Fig. 2a). The soluble expression of plant Rubisco in *E. coli* is approximately 2% CSP as measured by [^14^C]-CABP binding (Aigner et al. 2017). The ease of soluble expression and assembly, together with a very high carboxylation efficiency, make RPE Rubisco an outstanding candidate as a biological conduit for CO_2_ capture through synthetic biology. Emerging microbial platforms that can survive on CO_2_ as a sole carbon source (Gassler et al. 2020; Gleizer et al. 2019) under bioreactor conditions are ideal systems for RPE Rubisco as the atmospheric conditions can be user controlled. RPE Rubisco was previously reported as showing a poor specificity for substrate CO_2_ over O_2_ (Sc/o = 8.6) which is in line with the oxygenation sensitivity displayed by other Rubisco in the Form II clade (Robinson et al. 2003). Form II and Form III Rubiscos are universally poor at discriminating between CO_2_ and O_2_ as substrates and have low affinity for CO_2_ compared to the Form I clade (Davidi et al. 2020; Whitney et al. 2011; Yang et al. 2021). These features render them completely inappropriate for CO_2_ capture directly from air, and consequently, any relevance to photosynthetic improvement. However, as we show here, the CO_2_ assimilated in the form of d-lactate by *E. coli* harboring RPE Rubisco was 3.6-fold higher than that of 7002 Rubisco, which underscores its potential as a tool for microbial CO_2_ capture*.* Continued engineering of RPE Rubisco to further improve its carboxylation efficiency would push its kinetic profile further towards an increasingly optimal zone for high-efficiency microbial CO_2_ capture (Fig. 6e).

Analysis of the original host and habitat in which RPE Rubisco was discovered provides further physiological insights into its superior carboxylation activity and a potential resource for similar isoforms to explore. RPE Rubisco was identified from the chemolithoautotrophic symbiont in the trophosome of giant tubeworm *R. pachyptila* that lives near CO_2_-rich and O_2_-poor deep-sea hydrothermal vents (Robinson et al. 1998). *R. pachyptila* is probably the fastest-growing marine invertebrate (Lutz et al. 1994), but has no mouth or digestive tract. All organic carbon required to support its growth comes from the symbiont in its trophosome. The fast growth rate of the tubeworm in the sparsely populated deep sea can thus be partly explained by the superior CO_2_-fixation efficiency of RPE Rubisco. Rubiscos from similar trophosome and other endosymbionts inhabiting a range of host marine organisms that occupy similar environmental niches as *Riftia* provide may show similar utility for heterologous expression (Fig. 1b). These organisms exhibit frequent heterologous gene transfer (Li et al. 2018) which has likely adapted their Rubiscos enzymes to the remarkable yields observed in this study when expressed in *E. coli*. Interestingly, similar Rubisco found in the Eukaryotic endosymbionts (*Symbiodinium* and *Chromera*) of coral clade with the Rubisco in prokaryotic endosymbionts (Fig. 1b), further highlighting their unique adaptation for heterologous transfer, expression, and high-efficiency host CO_2_ assimilation.

RPE Rubisco was from chemolithoautotrophic microbes living in deep sea. Contrary to the general impression that (photo) autotrophic organisms or extremophiles always grow slowly, it is reported that the doubling times of *Thiomicrospira crunogena* and *Hydrogenomonas thermophila*, two chemolithoautotrophic microbes from deep-sea hydrothermal vents, were only about 1 h (Dobrinski et al. 2005) and 1.2 h (Takai et al. 2004), respectively. These doubling times were even faster than that of *Saccharomyces cerevisiae*, a well-known fast-growing heterotrophic microbe, indicating that the carbon fixation and metabolism in these chemolithoautotrophic microbes must be very efficient. Although photosynthesis by oxygenic photoautotrophs is the main contributor to CO_2_ fixation on earth, our results suggest that CO_2_ fixation by chemosynthesis of the chemolithoautotrophs might be more efficient and its potential may therefore be underestimated. A recent study (Davidi et al. 2020) also reported four Form II Rubisco (L_2_)_n_ variants with *k*_cat_^C^ higher than 7002 Rubisco, and three out of these four were also from chemolithoautotrophs, thus providing further evidence to our argument. There were no data on the heterologous expression and protein structure of these newly screened Form II Rubiscos in this report though (Davidi et al. 2020). It would thus be interesting to see whether these highly active Form II Rubiscos would be expressed/assembled in *E. coli* as well as RPE Rubisco, and whether they would exhibit similar potential for CO_2_ capture.

Previously, the in vivo Rubisco-based carbon fixation efficiency was evaluated by indirect calculation (Tseng et al. 2018; Yang et al. 2016). In these models, it was difficult to precisely evaluate Rubisco-based CO_2_ capture ability due to pyruvate decarboxylation after CO_2_ fixation. The Rubisco-based CO_2_ capture model developed in this work secures the fixed CO_2_ in the form of d-lactate, which is a carbon-conserving process. Using this model, we revealed that RPE Rubisco can help viable *E. coli* cells capture more CO_2_ than 7002 Rubisco. It should be pointed out that xylose can also be metabolized through the pentose phosphate pathway to produce d-lactate. 36% of xylose in strain RPE was consumed to the metabolic flux of the CO_2_-fixation bypass pathway. However, only 7.5% of xylose in strain 7002, which was one-fifth of that of the RPE Rubisco. This suggests the Rubisco activity determines both the speed and efficiency of CO_2_ capture.

Although there is great sequence and structural diversity among the Form I Rubiscos from oxygenic photoautotrophs and the Form II Rubiscos from chemolithoautotrophs, the two types of Rubiscos share some common features. The first one is the well-known trade-off between their carboxylation rate and affinity toward CO_2_. The most active Form I Rubisco from the oxygenic photoautotrophic cyanobacteria shows the lowest affinity toward CO_2_, with a *K*_M_^C^ in the range of 200–250 μM (Galmes et al. 2014; Hanson 2016; Whitney et al. 2011). RPE Rubisco, which has a higher carboxylation rate than the cyanobacterial Rubisco, also exhibits slightly higher CO_2_ affinity, with a *K*_M_^C^ of 172.4 μM. The second one is the CO_2_-rich surroundings. The Rubisco enzymes from oxygenic photoautotrophic cyanobacteria and C_4_ plants showing relatively high carboxylation activity but low CO_2_ affinity usually rely on a carbon concentrating mechanism. This also holds true for the RPE Rubisco from a chemolithoautotroph. It is reported that partial pressure of CO_2_ in the sea water was significantly elevated from 0.024 kPa in the vast region to 2.9 kPa around the tubeworm (Childress et al. 1993). Together with the reported high concentrations of carbonic anhydrase in the worm's plume and trophosome tissue, the internal total CO_2_ concentration of *R. pachyptila* can reach up to 31 mM (Childress et al. 1993). Under such a high internal CO_2_ concentration, the low affinity of RPE Rubisco toward CO_2_ will not limit its carboxylation efficiency. *E. coli* does not have a carbon concentrating mechanism, but the slightly higher CO_2_ affinity enabled *E. coli* harboring RPE Rubisco to capture CO_2_ under the supply of 10% CO_2_ in the environment. This implies *E. coli* harboring RPE Rubisco does not need a CO_2_-rich environment to play its carbon capture function.

It is generally known that extremely high expression of Rubisco is required in natural hosts to compensate for the shortage of its low carboxylation activity, which leads plant leaf protein content to consist up to 50% as Rubisco. Moreover, Rubisco from plant and cyanobacteria required their own specific chaperones to achieve such a high-level expression. The simple structure, high carboxylation efficiency, easy heterologous soluble expression/assembly characteristics of RPE Rubisco make it an interesting enzyme for further carbon fixation research. Additional improvement to the CO_2_ fixation rates in this study may be achieved by co-expression of RPE Rubisco activase genes (*cbb*O/*cbb*Q), as enzymatic deactivation in the absence of Rubisco activase has been shown to significantly reduce chemoheterotrophic Rubisco performance in heterologous systems (Gunn et al. 2020).

## Conclusion

The Form II RPE Rubisco from the endosymbiont of a deep-sea tubeworm *Riftia pachyptila* was identified and characterized in this study. RPE Rubisco has the potential to be used for carbon capture due to its higher carboxylation efficiency, easy heterologous soluble expression/assembly and simple hexamer structure. The CO_2_ assimilation efficiency of *E. coli* harboring RPE Rubisco was 3.6-fold higher than that of 7002 Rubisco using a designed CO_2_ capture model, demonstrating the application potential of RPE in microbial CO_2_ capture.

### Supplementary Information

**Additional file 1.** Additional Rubisco sequences information.

**Additional file 2.** Additional tables and figures.

## Data Availability

The data and the materials are all available in this article as well as the Additional files 1 and 2.

## Abbreviations

Rubisco: Ribulose-1,5-bisphosphate carboxylase/oxygenase
RPE Rubisco: The endosymbiont of *Riftia pachyptila* Rubisco
7002 Rubisco: *Synechococcus* sp. PCC7002 Rubisco
PRK: Phosphoribulokinase
CBB: Calvin–Benson–Bassham
RbcL: The large subunit gene of each selected Rubisco
CABP: 2-Carboxyarabinitol-1,5-diphosphate
RuBP: D-Ribulose-1,5-bisphosphate
3PGA: Glycerate-3-phosphate
Ribulose-5P: Ribulose-5-phosphate
F6P: Fructose-6-phosphate
G3P: Glyceraldehyde 3-phosphate
1,3BPG: 1,3-Bisphosphoglycerate
