# Supplementary material for: Discovery of a readily heterologously expressed Rubisco from the deep sea with potential for CO2 capture
Source: Bioresour Bioprocess. 2021 Sep 7;8(1):86. doi: 10.1186/s40643-021-00439-6 (PMC10992382; doi:10.1186/s40643-021-00439-6)
Supplement: Supplementary file 2 — Additional file 2. Additional tables and figures. [file 40643_2021_439_MOESM2_ESM.pdf]

Supporting Information

Discovery of a Readily-Heterologously-Expressed Rubisco from the Deep Sea with Potential for CO_2 Capture

Table S1. Strain and plasmid

Name	Plasmid/Strain	Description	Source
pET30a	pET30a		Lab storage
pET28a	pET28a		Lab storage
7002	pET30a-7002-PRK	Derived from pET30a. Inserting Rubisco encoding genes (rbcL-rbcX-rbcS) from *Synechococcus* sp. PCC7002 with P_{T7} –Ptrc double promoters each at the upstream of rbcL and rbcX into the *Nde* 1/*Xho* I sites; Inserting PRK encoding gene (*prk*) and the upsteam trpR-Ptrp promoter into the *Fsp* I/*Psh* AI sites	(Cai et al. 2014)
197	pET30a-RBC197-PRK	Derived from pET30a-7002-PRK. Inactivating Rubisco by a K197M mutation	(Cai et al. 2014)
197-2021	pET30a-RBC197-PRK 2021	Derived from pET30a-7002-PRK. Inactivating PRK by K20M and S21A mutation	(Cai et al. 2014)
RPE	pET30a-RPE-PRK	Rubisco from *Riftia pachyptila endosymbiont* was inserted into the *Nde* 1/*Xho* I sites of pET30a-7002-PRK	This work
RRU	pET30a-RRU-PRK	Rubisco from *Rhodospirillum rubrum* ATCC 11170 was inserted into the *Nde* 1/*Xho* I sites of pET30a-7002-PRK	This work
RPA	pET30a-RPA-PRK	Rubisco from *Rhodopseudomonas palustris* was inserted into the *Nde* 1/*Xho* I sites of pET30a-7002-PRK	This work
RCA	pET30a-RCA-PRK	Rubisco from *Rhodobacter capsulatus* was inserted to the *Nde* 1/*Xho* I sites of pET30a-7002-PRK	This work
MMA	pET30a-MMA-PRK	Rubisco from *Magnetospirillum magnetotacticum* was inserted into the *Nde* 1/*Xho* I sites of pET30a-7002-PRK	This work
RFE	pET30a-RFE-PRK	Rubisco from *Rhodoferax ferrireducens* T118 was inserted into the *Nde* 1/*Xho* I sites of pET30a-7002-PRK	This work
PNA	pET30a-PNA-PRK	Rubisco from *Polaromonas naphthalenivorans* CJ2 was inserted into the *Nde* 1/*Xho* I sites of pET30a-7002-PRK	This work
BL21(DE3)	BL21(DE3)	*F-, ompT, hsdS (rBB-mB-), gal, dcm (DE3)*	Novagen
BWLac	BWLac	BW25113Δ*frdABCD*Δ*pflB*::*ldhA*	Lab storage

Table S2. X-ray data collection and refinement statistics

**PDB ID**	**6IUS**
Wavelength	0.979
Space group	C 1 2 1
a,b,c (Å)	166.501, 107.842, 112.306
α,β,γ (°)	90, 130.04, 90
Resolution (Å)	50-2.12 (2.16-2.12)
No. of observations	586813
No. of unique reflections	86160
Completeness (%)	99.8 (99.8)
Overall I/ σ (*I*)	25.4
Last shll I/ σ (*I*)	3.4
Redundancy	6.8 (6.5)
R_{merge}	0.138 (0.396)
$R_{p.i.m}$	0.056 (0.166)
R_{work} /R_{free}	0.205/0.241
RMSD bond length (Å)	0.010
RMSD bond angle (°)	1.429
Ramachandran favored (%)	90.8
Ramachandran allowed (%)	8.8
Ramachandran outlier (%)	0.4

Table S3. Known solubilities of Rubisco in *E. coli* in the absence of additional factors.

Code	Form	Species	k_{cat} (s^{-1})	K_C (μM)	k_{cat}/K_C ($mM.s^{-1}$)	%CSP	Reference
1	I - 'green'	*A. thaliana*	3	9.8	306	0	(Aigner et al. 2017)
2	I - 'green'	*N. tabacum*	3.2	12.6	254	0	(Lin et al. 2020)
3	I - 'green'	*C. reinhardtii*	5.8	31	187	0	R. H. Wilson, unpublished.
4	I - 'green'	Synechococcus sp. PCC 7002	12.3	194.6	63	0.2	This study; (Emlyn-Jones et al. 2006)
5	I - 'green'	Synechococcus sp. PCC 6301	12.9	248	52	1	(Zhou et al. 2019)
6	I - 'green'	*T. elongatus*	7.8	104	75	6.6	(Wilson et al. 2018)
7	I - 'green'	*Candidatus P. breve*	2.2	22.2	99	7.5	(Banda et al. 2020)
8	I - 'red'	*G. monilis*	1.2	3.3	364	0	R. H. Wilson, unpublished.
9	I - 'red'	*G. sulphuraria*	2.6	9.3	280	0	R. H. Wilson, unpublished.
10	I - 'red'	*R. sphaeroides*	4.2	62	68	4.9	(Zhou et al. 2019)
11	III	*M. burtonii*	0.6	56.9	11	7	(Wilson et al. 2016)
12	II	*R. rubrum*	12.3	149	83	0.2	(Zhou et al. 2019)
13	II	*R. pachyptila*	16.4	172.4	95	11.7	This study

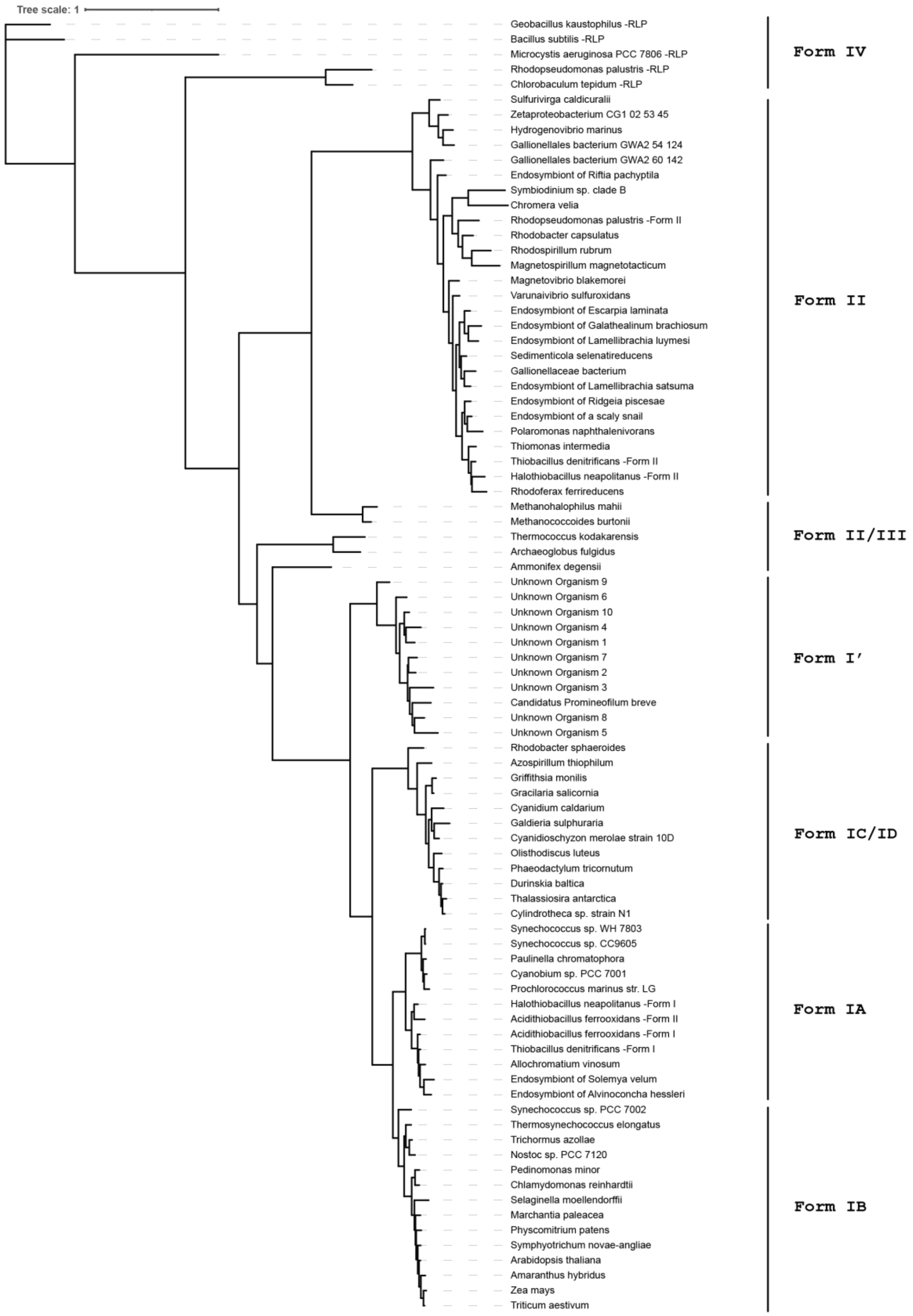

Figure S1. Maximum likelihood phylogenetic tree of the Rubisco large subunit (RbcL) protein sequences enriched for sequences from autotrophic endosymbionts.

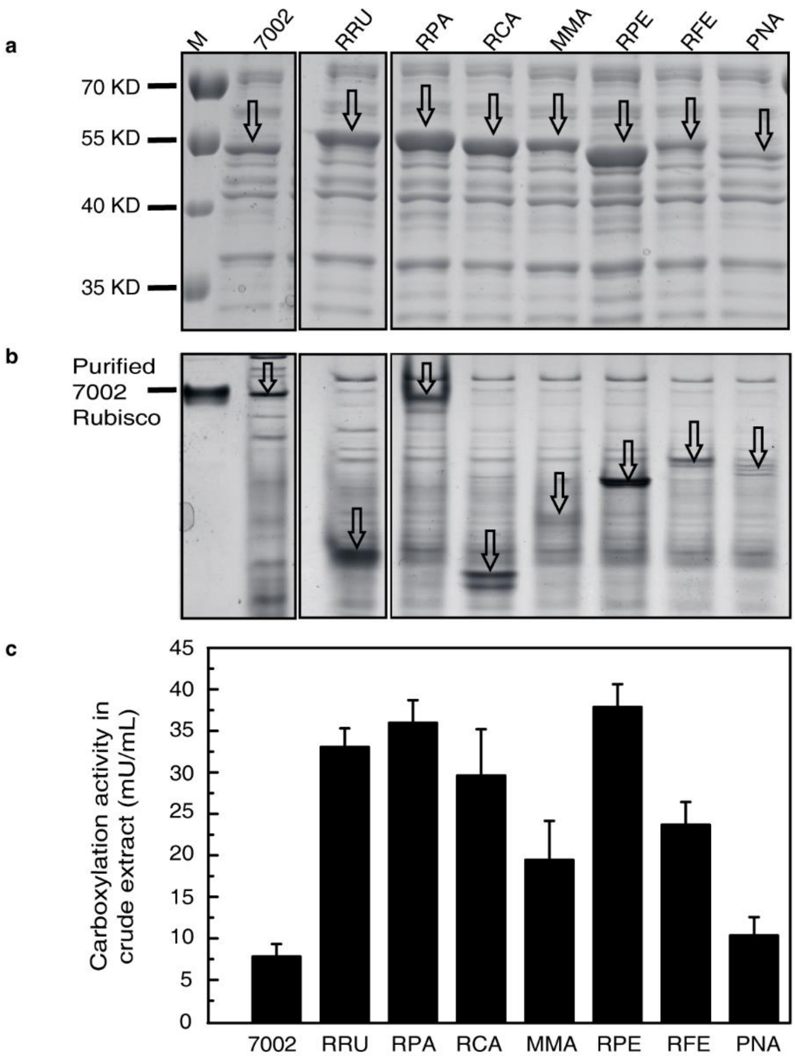

Figure S2. Expression, assembly and crude carboxylation activity of Form II Rubisco in *E. coli* by using crude cell extracts. 7002, *Synechococcus* PCC7002; RRU, *Rhodospirillum rubrum* ATCC 11170; RPA, *Rhodopseudomonas palustris*; RCA, *Rhodobacter capsulatus*; MMA, *Magnetospirillum magnetotacticum*; RPE, *Riftia pachyptila endosymbiont*; RFE, *Rhodoferax ferrireducens* T118; PNA, *Polaromonas naphthalenivorans* CJ2 (Table S1). All Rubisco were expressed in *E. coli* BL21 (DE3) by IPTG induction. Soluble proteins in the crude cell extracts were subjected by **(a)** SDS-PAGE (12%, w/v), **(b)** native-PAGE (8%, w/v), **(c)** carboxylation activity assay using $NaH^{13}CO_3$. Arrows indicated the bands of Rubisco. The mean values and standard derivations of three independent assays were shown.

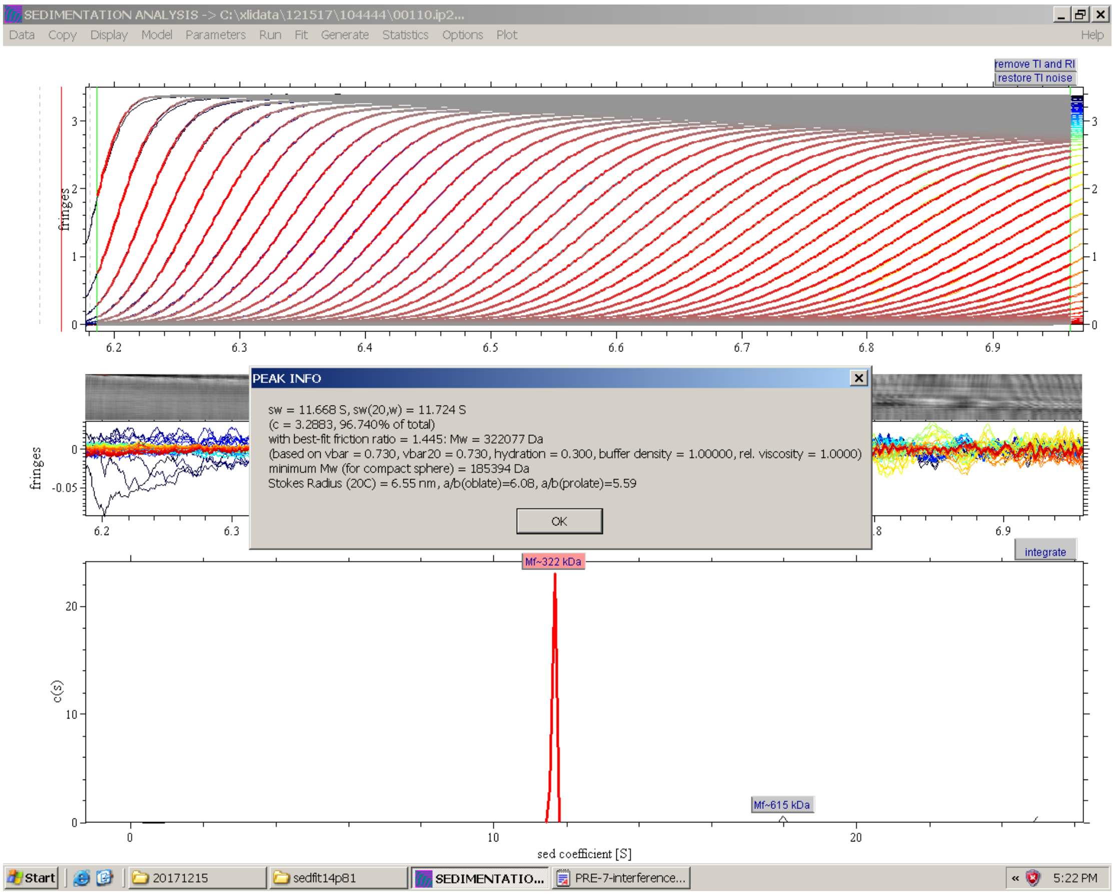

Figure S3. Analytic ultracentrifugation of RPE Rubisco. The sedimentation velocity of RPE Rubisco was determined using Proteome Lab XL-I analytical ultracentrifuge (Beckman Coulter, Brea, CA) equipped with an AN-60Ti rotor and the conventional double-sector aluminum centerpieces of 12 mm optical path length. A mixture of 380 μL of purified RPE Rubisco and 400 μL of buffer (20 mM HEPES, pH8.0, 4 mM $MgCl_2$, 2 mM KCl, 0.2 mM EDTA, 2 mM DTT) were loaded and centrifuged at 20°C and 26,000 rpm. The continuous scan mode and radial spacing of 0.003 cm were used. The wavelength for absorbance was 280 nm. Scans were collected at every 3 min intervals. The fitting of absorbance versus cell radius data was performed using SEDFIT software (https://sedfitsedphat.nibib.nih.gov/software) and continuous sedimentation coefficient distribution c(s) model, covering range of 0-15 S. Biophysical parameters of the buffer and protein were set as below: density ρ = 1.0000 g/cm3, viscosity η = 0.01002, partial specific volume of protein V-bar = 0.73000 cm3/g.

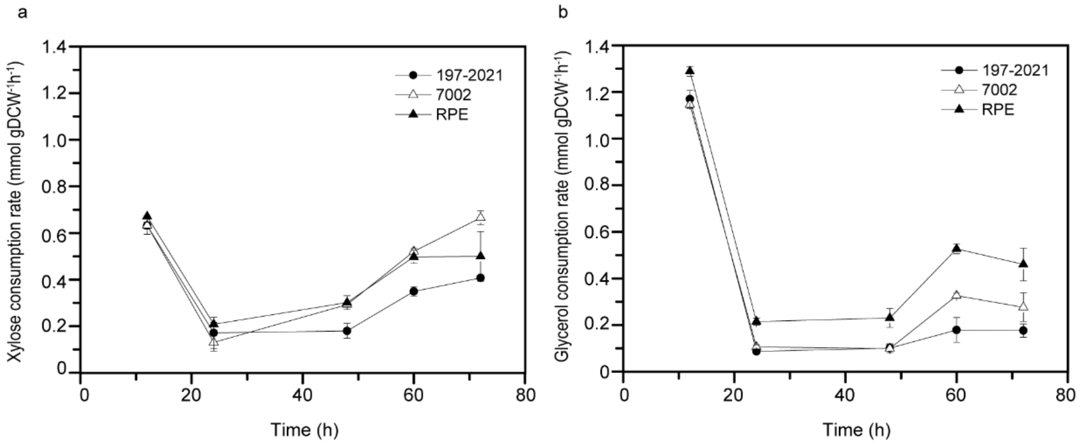

Figure S4. Xylose (a) and glycerol (b) consumption rates during D-lactate production.

Specific substrate consumption rates were calculated using the minusing between two adjacent sampling points using the data presented in Fig. 6a (xylose), Fig. 6b (glycerol). An equation of one unit OD_{600}= 0.3 gDCW/L (Soini et al. 2008) was used to convert the OD into dry cell weight. The OD_{600} of samples remained around 2 as described in the materials and method.

References

Aigner H, Wilson RH, Bracher A, Calisse L, Bhat JY, Hartl FU, Hayer-Hartl M (2017) Plant RuBisCo assembly in *E. coli* with five chloroplast chaperones including BSD2. Science 358: 1272-+. dol:10.1126/science.aap9221.

Banda DM, Pereira JH, Liu AK, Orr DJ, Hammel M, He C, Parry MAJ, Carmo-Silva E, Adams PD, Banfield JF, Shih PM (2020) Novel bacterial clade reveals origin of form I Rubisco. Nat Plants 6: 1158-66. dol:10.1038/s41477-020-00762-4.

Cai Z, Liu GX, Zhang JL, Li Y (2014) Development of an activity-directed selection system enabled significant improvement of the carboxylation efficiency of Rubisco. Protein Cell 5: 552-62. dol:10.1007/s13238-014-0072-x.

Emlyn-Jones D, Woodger FJ, Price GD, Whitney SM (2006) RbcX can function as a Rubisco chaperonin, but is non-essential in *Synechococcus* PCC7942. Plant Cell Physiol 47: 1630-40. dol:10.1093/pcp/pcl028.

Lin MT, Stone WD, Chaudhari V, Hanson MR (2020) Small subunits can determine enzyme kinetics of tobacco Rubisco expressed in *Escherichia coli*. Nature Plants. doi:10.1038/s41477-020-00761-5.

Soini J, Ukkonen K, Neubauer P (2008) High cell density media for *Escherichia coli* are generally

designed for aerobic cultivations - consequences for large-scale bioprocesses and shake flask cultures. Microb Cell Fact 7: 26. doI:10.1186/1475-2859-7-26.
Wilson RH, Alonso H, Whitney SM (2016) Evolving *Methanococcoides burtonii* archaeal Rubisco for improved photosynthesis and plant growth. Sci Rep-Uk 6. doI:10.1038/srep22284.
Wilson RH, Martin-Avila E, Conlan C, Whitney SM (2018) An improved *Escherichia coli* screen for Rubisco identifies a protein-protein interface that can enhance CO_2-fixation kinetics. J Biol Chem 293: 18-27. doI:10.1074/jbc.M117.810861.
Zhou Y, Whitney S (2019) Directed evolution of an improved Rubisco; In vitro analyses to decipher fact from fiction. Int J Mol Sci 20. doI:10.3390/ijms20205019.
